# *Ralstonia solanacearum* type III effector RipAA targets chloroplastic AtpB to modulate an incompatible interaction on *Nicotiana benthamiana*

**DOI:** 10.3389/fmicb.2023.1179824

**Published:** 2023-05-18

**Authors:** Yangchen Miao, Leilei Wu, Qi Xue, Qiuyan Zhang, Huasong Zou

**Affiliations:** School of Life Sciences and Health, Huzhou College, Huzhou, China

**Keywords:** *Ralstonia solanacearum*, RipAA, hypersensitive response, chloroplastic AtpB, incompatible interaction

## Abstract

**Introduction:**

The type III effector RipAA of *Ralstonia solanacearum* GMI1000 plays a critical role in the incompatible interaction on *Nicotiana benthamiana*.

**Methods:**

The RipAA was transiently expressed in *N. benthamiana* by Agrobacterium-mediated transformation. Chemical staining with trypan blue and DAB were conducted to examine the cell death and the accumulation of hydrogen peroxide (H_2_O_2_), respectively. The expression of the marker genes for salicylic acid (SA) and jasmonic acid (JA) signaling was evaluated by quantitative reverse transcription PCR (qRT-PCR). The proteins interacted with RipAA was identified from *N. benthamiana* by yeast two-hybrid and pull-down assays. A TRV-mediated gene silencing was used to assess the role of host gene in response to RipAA expression and *R. solanacearum* infection.

**Results and discussion:**

RipAA induced the accumulation of hydrogen peroxide (H_2_O_2_) and genome DNA degradation in *N. benthamiana*, which were accompanied by a hypersensitive reaction. Simultaneously, the marker genes for salicylic acid (SA) signaling were induced and those for jasmonic acid (JA) signaling were reduced. *N. benthamiana* chloroplastic AtpB, the ATPase β subunit, was identified as an interactor with RipAA. The silencing of *atpB* in *N. benthamiana* resulted in the inability of RipAA to induce a hypersensitive response, a compatible interaction with GMI1000, and an enhanced sensitivity to bacterial wilt. Our data support the concept that RipAA determines host-range specificity by targeting the host chloroplastic AtpB.

## 1. Introduction

Bacterial wilt disease is caused by a species complex of *Ralstonia solanacearum* (*Rso*), which poses a severe threat to a wide range of hosts, including numerous solanaceous plants (Paudel et al., 2020). *Rso* isolates from different regions around the world are highly virulent and exhibit a high degree of genetic diversity (Lebeau et al., 2011). In particular, the isolates show varied pathotypes in tobacco, eggplant (*Solanum melongena*), tomato (*S. lycopersicum*), potato (*S. tuberosum*), and peanut (*Arachis hypogaea* Linn.) (Buddenhagen et al., 1962; He et al., 1983). The varied pathotypes are genetically determined by the type III secretion system (T3SS)-delivered effectors of the pathogen. In the first sequenced reference strain GMI1000 (Salanoubat et al., 2002), 71 effectors have been identified by the use of a number of transcriptional and translocational analyses (Cunnac et al., 2004; Occhialini et al., 2005; Peeters et al., 2013). One effector RipP2 (formerly designated PopP2) specifies the avirulent property on *Arabidopsis thaliana* ecotype Nd-1 by interacting with RRS1-R, which confers resistance to bacterial wilt (Deslandes et al., 2003). The YopJ/AvrRxv family effector RipP1 (formerly designated PopP1) is involved in the incompatible interaction with petunia plants. The disruption of *ripP1* gene in GMI1000 results in a compatible interaction in petunia (Lavie et al., 2002).

The avirulence of GMI1000 toward tobacco species is jointly determined by two T3SS effectors RipP1 and RipAA (formerly designated AvrA) (Poueymiro et al., 2009; Chen et al., 2018). Even though the transient expression of either *ripP1* or *RipAA* induces a hypersensitive reaction (HR) in tobacco species, RipAA plays a major role in its avirulent properties (Robertson et al., 2004; Poueymiro et al., 2009). In contrast to the wild type GMI1000, the *ripAA* mutant generates wilt-like symptoms when infiltrated into *N. tabacum* leaves (Robertson et al., 2004; Poueymiro et al., 2009). The function of *ripAA* was discovered from a genetic locus from *Rso* strain AW1 in a 2-kb DNA fragment that determined an incompatibility at the species level of tobacco (Carney and Denny, 1990). The wild type RipAA is necessary for an *Rso* population in the southeastern US to induce HR or avirulence on tobacco, since the isolates from diseased tobacco plants possess mutated RipAA alleles that fail to induce HR (Robertson et al., 2004). In contrast, the double deletion of *ripAAripP1* has no distinctive effect on the induction of HR in Japanese strains 8107 and MAFF211471, suggesting that there are other unknown avirulent determinants (Chen et al., 2018). In the Japanese strain RW1000 that is avirulent on tobacco, RipB acts as a major avirulence factor in *Nicotiana* species but not with RipAA or RipP1 (Nakano and Mukaihara, 2019).

Plant chloroplast F1F0-ATP synthase (cpATPase) represents a thylakoid membrane-associated protein complex composed of a membrane-spanning F0 and a soluble F1, which utilizes the proton motive force across the thylakoid membrane to drive ATP biosynthesis (Rühle and Leister, 2015). During the sequential assembly of cpATPase, subunit b is involved in the formation of a peripheral region that cooperates with a central stalk composed of subunits γ and ε (Hahn et al., 2018). The *atpB* gene for cpATPase is located in the chloroplast chromosome that encodes the β-subunit of ATP synthase and is controlled by the nuclear-encoded factors for transcriptional expression (Drapier et al., 1992). Even though chloroplastic *atpB* is usually utilized as a molecular marker for phylogenetic analysis and genetic diversity (Refulio-Rodriguez and Olmstead, 2014; Chen et al., 2020), its biological function is undoubtedly the contribution for ATPase activity owing to a few special features, such as membrane binding, ATP binding and nucleotide hydrolyzing motifs (Mills and Richter, 1991; Chen et al., 1992). The level of expression of AtpB in chloroplast organelles varies in common bean upon drought stress treatment, suggesting a disorder of ATP synthesis and photosynthetic processes in this circumstance (Zadražnik et al., 2019). Furthermore, the chloroplastic AtpB is closely related to plant-pathogen interactions (Megias et al., 2018; Farias and Giorello, 2020). The abundance of chloroplastic AtpB significantly increases in the chloroplast when *N. benthamiana* is infected with *Tomato blistering mosaic virus* (Megias et al., 2018). Direct evidence originates from the fact that the viral protein TGB1_*L*88_ of *Alternanthera mosaic virus* (AltMV) is selectively bound by chloroplastic AtpB to elicit a defense response on *N. benthamiana* (Seo et al., 2014). The role of host chloroplastic *atpB* in response to *Rso* infection has not yet been elucidated.

This research aimed to discover the relevant mechanisms utilized by RipAA to modulate interaction with its host in *Rso*. In addition to the new findings on physiological changes, the chloroplastic AtpB was identified as a target of RipAA to modulate the incompatible interaction between GMI1000 and *N. benthamiana.* Those data provide new knowledge on the molecular events in association with the host specificity of *Rso*.

## 2. Materials and methods

### 2.1. Plant, bacterial, and plasmid materials

*Nicotiana benthamiana*, *N. tabacum* cv. NC89, and *Capsicum annuum* cv. Yanshan01 seeds were germinated and cultivated in a greenhouse under a 16/8 h light/dark photoperiod at 25°C. All the strains and plasmids used in this study are listed in Supplementary Table 1. *Escherichia coli* and *Agrobacterium tumefaciens* were cultivated in Luria-Bertani media at 37°C and 28°C, respectively. *Rso* GMI1000 and FJ1003 were grown at 28°C in nutrient-rich broth media (Sun et al., 2020). *Saccharomyces cerevisiae* strain AH109 for the yeast two-hybrid analysis was cultured in YPD media (1% yeast extract, 2% peptone, and 2% glucose) at 30°C.

### 2.2. Examination of the hypersensitive response induced by RipAA

The coding sequence of *ripAA* was amplified by high-fidelity PCR polymerase with RipAA-*Hin*dIII-F and RipAA-*Pst*I-R primers (Supplementary Table 2), and subsequently cloned into the pHB vector at *Hin*dIII and *Pst*I cleavage sites. The truncated mutants (RipAA_1–216_, RipAA_1–196_, RipAA_1–166_, RipAA_1–126_, and RipAA_1–89_) were cloned into the pHB vector using the same cloning strategy. The constructs were individually transferred into *A. tumefaciens* strain GV3101 for transient expression assays. The cultured GV3101 was suspended in infiltration media (10 mM MgCl_2_, 10 mM MES, and 200 μM acetosyringone). The GV3101 cells were infiltrated into *N. benthamiana* leaves with a needleless syringe and incubated at room temperature for 2 h. Macroscopic HR was scored at 48 h post-infiltration. Cell death and hydrogen peroxide (H_2_O_2_) accumulation were assayed by trypan blue and 3,3′-diaminobenzidine (DAB) staining, respectively, (Sun et al., 2020). The DNA was extracted using a Plant Genomic DNA Kit (Tiangen, Beijing, China), and separated by electrophoresis on a 1.0% (w/v) agarose gel in the presence of 1 μL/mL nucleic acid dye GeneGreen. Each experiment was repeated three times.

### 2.3. Quantitative real-time PCR

Total RNA was extracted from *N. benthamiana* using a Plant RNA Kit (Omega, Shanghai, China). After quality examination and cDNA synthesis (Sun et al., 2020), quantitative real-time PCR (qRT-PCR) experiments were conducted to detect the levels of expression of defense genes using the following parameters: denaturation at 95°C for 30 s, followed by 40 cycles of 95°C for 5 s and 55°C for 20 s. The expression of *EF1*α was used as an internal control (Supplementary Table 2). Relative levels of expression and statistical analyses were conducted using CFX Maestro software (Bio-Rad, Hercules, CA, United States). Relative fold changes in gene expression were normalized against the expression of *EF1*α using the 2^–Δ^ ^Δ^ *^C^*^*t*^ threshold method. Each experiment was conducted in triplicate and repeated three times.

### 2.4. Subcellular localization assay

To study the subcellular localization in *N. benthamiana*, RipAA was fused to GFP in pGDG and RFP in pGD3Gmcherry. The GFP-RipAA and RFP-RipAA fusions were expressed in *N. benthamiana* by *A. tumefaciens*-mediated transient expression assays. Leaf disks were harvested for fluorescence signal examination using a Leica confocal laser scanning microscope (SP8; Leica, Wetzlar, Germany). Western blotting was performed to detect protein expression in *N. benthamiana* according to a previously described method (Sun et al., 2020). For the subcellular localization of YFP-RipAA in *A. thaliana* protoplasts, RipAA was cloned in pICH47811 fused with YFP. At 8 h post-transfection with YFP-RipAA using a PEG-mediated method, the protoplasts were subjected to fluorescence signal examination. For each construct or transformation analysis, three different samples were examined. The experiments were conducted in triplicate.

### 2.5. Assays for the plant response to bacterial wilt

The plant response to bacterial wilt was determined from the disease severity and percent severity index. *Rso* cells were prepared to a final concentration of 1 × 10^8^ CFU/mL. The symptoms on leaves were viewed by infiltrating 200 μL of an *Rso* cell suspension into the expanded leaves, and the wilt was scored 3 days post-inoculation. For the petiole inoculation method, 3 μL of bacterial suspension was inoculated onto a freshly cut wound on the petiole surface (Hu et al., 2019). For soil soaking inoculation, the plant roots were first wounded and then inoculated with 10 mL of an *Rso* cell suspension per pot (Sun et al., 2020). The inoculated plants were placed at 28°C to assess disease severity and calculate the percent severity index. The calculation for this index was recorded on a 0 to 4 disease severity scale as follows: 0 = no wilting; 1 = 1–25% of leaves wilted; 2 = 26–50% of leaves wilted; 3 = 51–75% of leaves wilted, and 4 = 76–100% of leaves wilted. The experiments were repeated four times. Each replicate contained 10 plants per treatment.

### 2.6. Yeast two-hybrid assay

The construct pGBKT7:RipAA was used as bait to screen interacting proteins from a cDNA library of *N. benthamiana.* The cDNA pool and pGBKT7:RipAA plasmid were co-transformed into the yeast host strain AH109 using standard lithium acetate-mediated transformation. Following a positive interaction that was identified as chloroplastic AtpB based on DNA sequencing, the complete ORF of chloroplastic AtpB was cloned into pGBKT7 to verify that pGADT7 harbored the factor responsible for the interaction with RipAA (Supplementary Table 1). The interaction between NbDDX3 (pGADT7:NbDDX3) and SDE1 (pGBKT7:SDE1) was used as a positive control (Zhou et al., 2020). The yeast two-hybrid experiment was conducted in triplicate.

### 2.7. GST pull down assay

The full-length coding region of *atpB* was cloned into pET41a(+) at *Eco*RI*-Sal*I sties, and RipAA was cloned into pMAl-C4X at *Eco*RI*-Pst*I sites (Supplementary Table 1). GST-AtpB and MBP-RipAA were then expressed in *Escherichia coli* BL21 (DE3) by induction with 1.0 mM isopropyl-β-D-thiogalactopyranoside (IPTG). GST-AtpB and MBP-RipAA were purified as previously described (Wu et al., 2019). GST and GST-AtpB were separately incubated with MBP-RipAA at 4°C for 60 min, and the final elution was subjected to a Western blot assay with an MBP antibody to verify the interaction between RipAA and chloroplastic AtpB. The experiment was conducted in triplicate.

### 2.8. Co-immunoprecipitation and mass spectrometry

To generate the GFP-RipAA fusion protein, RipAA was cloned in the pGDG vector with pGDGRipAA.F and pGDGRipAA.R primers at the *Bgl*II and *Sal*I sites (Supplementary Table 2), which generated a GFP-RipAA fusion driven by the 35S promoter. GFP-RipAA was transiently expressed in *N. benthamiana* leaves by the mediation of *A. tumefaciens* GV3101. At 36 h post-infiltration, 0.2 g leaves were fully ground in liquid nitrogen and then incubated for 30 min at 4°C in 200 μL lysis buffer (10 mM Tris/HCl pH 7.5, 150 mM NaCl, 0.5 mM EDTA, 0.5% Non-idet™ P40 Substitute, and 1 μL protease inhibitor). Followed by centrifugation at 10,000 × *g* for 2 min to remove debris, the cell lysate was mixed with 300 μL dilution buffer (10 mM Tris/HCl pH 7.5, 150 mM NaCl, and 0.5 mM EDTA). The mixture was incubated for 2 h at 4°C on a rotary mixer with GFP-trap beads that had been equilibrated with ice-cold dilution buffer. Followed by a triple wash with buffer (10 mM Tris/HCl pH 7.5, 150 mM NaCl, and 0.05% Non-idet Buffernidet™ P40 Substitute), the GFP-trap beads were resuspended in 2 × SDS-sample buffer and boiled for 10 min to dissociate bound proteins. The protein samples that were generated by GFP-trap immunoprecipitations were analyzed by 12% SDS-PAGE. Specific bands in the leaf extracts that expressed GFP-RipAA compared with the GFP control were excised from the gel and then subjected to a mass spectrometry analysis by Huada Gene Technology Co., Ltd. (Shenzhen, China). The proteins with >30% average coverage from three repeats were considered as putative interacting candidates.

### 2.9. Virus-induced gene silencing

The virus-induced gene silencing was performed using TRV2:*NbPDS* as the control to evaluate the silencing efficiency. A 404-bp partial sequence of *N. benthamiana* chloroplastic *atpB* was inserted into the pTRV2 vector to knock down *atpB* expression. TRV2:*gfp* that harbored a 358-bp fragment of the *gfp* gene was used as the control (Sun et al., 2020). A mixture of GV3101 cultures (1:1, v/v) that contained pTRV1 and pTRV2 constructs was co-infiltrated into 20-day-old *N. benthamiana* leaves. When the *PDS*-silenced plants exhibited a photo-bleached phenotype, qRT-PCR was performed to detect the transcript level of chloroplastic *atpB* in the silenced plants. Next, RipAA was transiently expressed by the infiltration of GV3101 at OD_600_ = 0.2, 0.1, and 0.05. The macroscopic HR reaction was recorded 2 days post-agroinfiltration. The silencing experiments were repeated four times, with five independent plants for each repeat.

### 2.10. Sequence and data analysis

The complete coding sequence of *N. benthamiana* chloroplastic AtpB was obtained by a BLAST search in GenBank. Chloroplastic AtpB homologs in representative solanaceous plants were retrieved from the Solanaceae Genomics Network.^1^ Secondary and 3-D structural modeling were analyzed with RaptorX Structure Prediction.^2^ Transmembrane domain prediction was performed with MINNOU.^3^ The evolutionary distances of 12 chloroplastic AtpB homologs were inferred using the Maximum Likelihood method based on the JTT matrix-based model in MEGA5 (Tamura et al., 2011). When the number of common sites was <100 or <one fourth of the total number of sites, the maximum parsimony method was used. Otherwise, the BIONJ method with an MCL distance matrix was used. The statistical analyses were conducted with SPSS 17.0 (SPSS, Inc., Chicago, IL, United States). Significant differences were assessed using a one-way analysis of variance (ANOVA) or student’s *t*-test.

## 3. Results

### 3.1. RipAA induces physiological changes and differential expression of signaling marker genes in *N. benthamiana*

**FIGURE 1:**
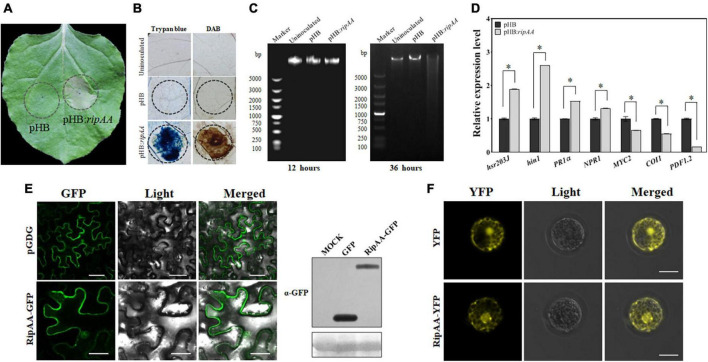
Expression of RipAA in the plant. **(A)** Macroscopic hypersensitive response in *Nicotiana benthamiana* induced by RipAA at 2 days post-agroinfiltration. The empty vector pHB was used as the negative control in *Agrobacterium*-mediated transformation. The infiltration areas are indicated by circles. **(B)** Histochemical staining of cell death and hydrogen peroxide. Cell death and hydrogen peroxide accumulation were revealed by trypan blue and DAB staining, respectively. The infiltration areas are indicated by circles. **(C)** Agarose gel electrophoretic analysis of DNA degradation. The DNA was extracted from leaves at 12 and 36 h post-agroinfiltration. **(D)** qRT-PCR analysis of salicylic acid and jasmonic acid signaling marker genes. The transcript level of each gene in plants transformed with empty vector pHB was set to 1, and the levels in the plant that transformed *ripAA* were calculated relative to that. Data represent the mean and standard deviation of three technical replicates (student’s *t*-test, *P < 0.05). All the experiments were repeated three times 36 h post-agroinfiltration. **(E)** Subcellular localization of RipAA-GFP in *N. benthamiana* cells. The samples were examined under a confocal microscope 36 h after agroinfiltration. All the experiments were conducted in triplicate. The scale bar represents 50 μm. The gels on the right show the expression of GFP and RipAA-GFP in a western blot using α-GFP antibody. **(F)** Subcellular localization of YFP-RipAA in *Arabidopsis thaliana* protoplasts. Yellow fluorescent protein fluorescence images were recorded at 8 h post-transfection. Scale bars of all the images represent 20 μm. DAB, 3,3′-diaminobenzidine; GFP, green fluorescent protein; qRT-PCR, real-time quantitative reverse transcription PCR.

The plants infiltrated with GV3101 that harbored pHB:*ripAA* exhibited a strong visual phenotype of the HR reaction at 48 h post-infiltration (Figure 1A). The cell death was verified from the infiltration areas by trypan blue staining, and the accumulation of H_2_O_2_ was revealed by DAB staining (Figure 1B). The transient expression of RipAA additionally induced the degradation of nuclear DNA in *N. benthamiana* (Figure 1C). To examine the molecular changes mediated by RipAA, the marker genes for cell death, salicylic acid (SA), and jasmonic acid (JA) signaling were studied. In comparison with the control, the transcript levels of the cell death markers *hsr203J* and *hin1* were induced by 1.9-and 2.6-fold, respectively. The SA signaling marker gene *PR1a* was induced 1.5-fold, and *NPR1* was induced 1.3-fold. In contrast, the JA signaling markers *COI1*, *MYC2* and *PDF1.2* were significantly suppressed by the expression of RipAA. The transcript level of *PDF1.2* was reduced by 80% compared with the control (Figure 1D).

To determine the subcellular localization, RipAA was fused to the C-terminus of green fluorescent protein (GFP) in the pGDG vector. *Agrobacterium*-mediated transient expression revealed that GFP-RipAA was localized to the cell membrane, cytoplasm, and nucleus in *N. benthamiana* cells (Figure 1E). When RipAA was fused with a red fluorescent protein (RFP), a similar location of RFP-RipAA was found in *N. benthamiana* (Supplementary Figure 1). To confirm the localization of RipAA in plant cells, a yellow fluorescent protein (YFP)-RipAA fusion was transformed into *A. thaliana* protoplasts. As expected, the YFP fluorescence was identified in the nucleus, membrane, and cytoplasm (Figure 1F).

### 3.2. C-terminus is necessary for RipAA to induce HR

**FIGURE 2:**
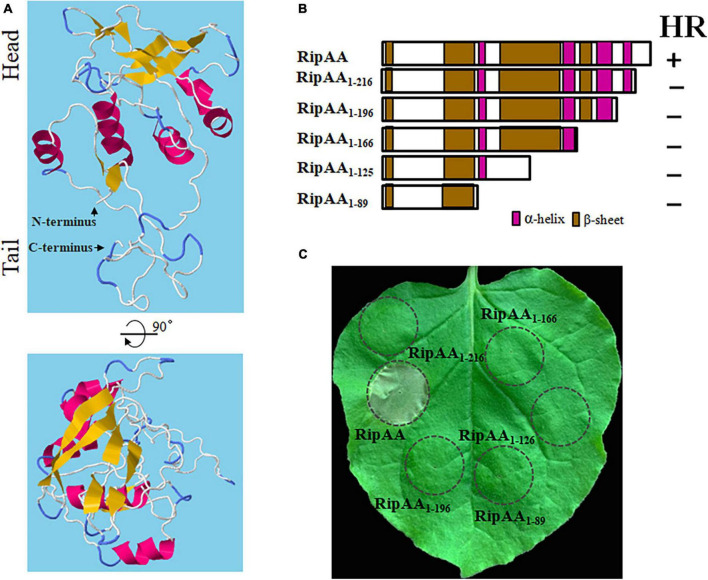
Necessity of the C-terminus of RipAA in the induction of a HR. **(A)** A 3-dimensional structure of RipAA predicted by RaptorX. α-helix structures are shown in red, and ß-sheet structures are shown in brown. **(B)** Schematic map representing RipAA truncated mutants and their abilities to induce a hypersensitive response. The mutants were cloned in binary vector pHB and expressed in *Nicotiana benthamiana* by *Agrobacterium*-mediated transient transformation. The locations of α-helix and β-sheet domains are shown in red and brown, respectively. **(C)** The loss of hypersensitive response induction by RipAA mutants in *N. benthamiana*. Agroinfiltration areas are indicated by circles. Macroscopic hypersensitive response phenotypes were recorded 2 days post-agroinfiltration. The experiments were conducted in triplicate, and repeated three times. HR, hypersensitive response.

RipAA contains 230 amino acids with a 58-amino acids T3SS signal at its N-terminus. No typical transmembrane domain was located by means of membrane protein prediction. However, five small α-helices and nine small β-sheet structures were observed, followed by second and tertiary structure analysis on RaptorX (see text footnote 2). The positions of five α-helices are 81–86, 89–94, 156–165, 184–196, and 208–216. Nine β-sheets are located at positions 2–9, 56–59, 62–63, 69–71, 74–76, 109–115, 121–127, 167–179, and 199–200 (Figure 2A). As suggested by the model from Raptor analysis, the three-dimensional (3-D) structure of RipAA shows distinctive head, intermediate and tail partitions. Most β-sheets are spatially located in the head sections adjacent to each other to form a plane surface. Most α-helices are located at intermediate sections and serve as a connector between the head and tail. The tail structure is composed of N- and C-termini, each containing a small β-sheet structure (2–9 and 199–200) (Figure 2A). Based on the locations of α-helices and β-sheets across the whole protein, RipAA clearly contains four α-helix and four β-sheet domains (Figure 2B). Based on this structural feature, a series of truncated RipAAs in which diverse C-terminal sequences had been deleted were constructed to examine the induction of HR in *N. benthamiana* (Figure 2B). The results showed that the C-terminus was absolutely required for RipAA to induce HR. Deletion of the last 14 amino acids at the C-terminus resulted in a complete loss of the ability to induce HR (Figure 2C).

### 3.3. RipAA interacts with plant chloroplastic AtpB

**FIGURE 3:**
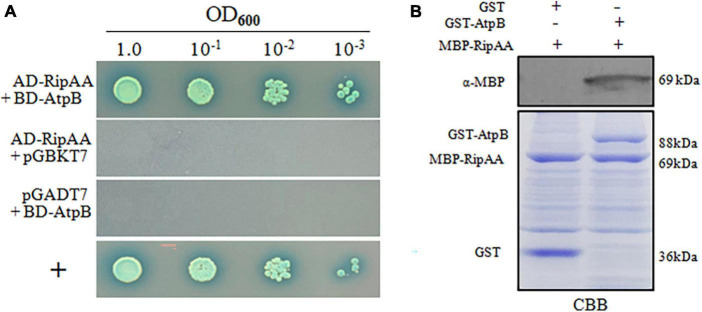
Identification of RipAA-interacting chloroplastic AtpB protein from *Nicotiana benthamiana*. **(A)** The yeast isolate AH109 that harbored AD-RipAA (pGADT7:RipAA) and BD-AtpB (pGBKT7:AtpB) was grown on SD/-Ade/-Leu/-Trp/-His/plates. AH109 cells were adjusted to the density of OD_600_ = 1.0 and diluted 10-fold in series. For each series, 2 μL suspensions were incubated on plates that were supplied with 20 μg/mL X-α-gal for 4 days. The interaction between NbDDX3 and SDE1 was used as a positive control (+). The Y2H assays were repeated three times. **(B)** GST pull-down assays that show the interaction between RipAA and chloroplastic AtpB. MBP-RipAA and GST-AtpB were incubated overnight at 4°C with 300 μL of glutathione resin. The final eluted protein samples were separated on a 12% SDS-PAGE gel and immunoblotted with anti-MBP. The negative control included the incubation of GST protein with MBP-RipAA. The protein abundance was revealed by CBB staining. Similar results were obtained from three independent repeats. CBB, coomassie brilliant blue.

In yeast two-hybrid (Y2H) assays, two positive clones were obtained from the *N. benthamiana* cDNA library using RipAA as bait. After the pGADT7 plasmids were extracted from yeast cells, Y2H assays were performed to repeat the positive interaction. DNA sequencing revealed that the two plasmids are duplicates and correspond to the chloroplastic *atpB* gene that encodes the chloroplastic AtpB. Subsequently, the entire 1494-bp coding sequence of chloroplastic *atpB* was cloned into the pGBKT7 plasmid to repeat the Y2H performances with pGADT7-RipAA (Figure 3A). A glutathione S-transferase (GST) pull-down assay was conducted to further verify the physical interaction between RipAA and chloroplastic AtpB. GST-AtpB was used as the bait and incubated with MBP-RipAA. Compared with the control sample from the GST tag, GST-AtpB successfully pulled down MBP-RipAA (Figure 3B).

To examine the interaction *in vivo*, a co-immunoprecipitation (co-IP) analysis was applied on *N. benthamiana* that transiently expressed GFP-RipAA. The expression of GFP-RipAA was examined under confocal laser microscopy at 36 h post-inoculation. A GFP affinity column was then loaded with total protein extracts from GFP-RipAA for a co-immunoprecipitation analysis. The specific protein complex was subjected to mass spectrometric analyses. Eight proteins associated with the chloroplastide were identified, including the chloroplastic AtpB, ribulose bisphosphate carboxylase large chain, ATP synthase alpha subunit, phosphoglycerate kinase, carbonic anhydrase, glyceraldehyde-3-phosphate dehydrogenase A, ribulose bisphosphate carboxylase/oxygenase activase 1 and ribulose bisphosphate carboxylase/oxygenase activase 2 (Supplementary Table 3). The chloroplastic AtpB had the highest coverage at 67%, supporting the hypothesis that chloroplastic AtpB directly interacts with RipAA *in vivo*.

### 3.4. Knock-down of chloroplastic *atpB* in *N. benthamiana* results in a compatible interaction with GMI1000

**FIGURE 4:**
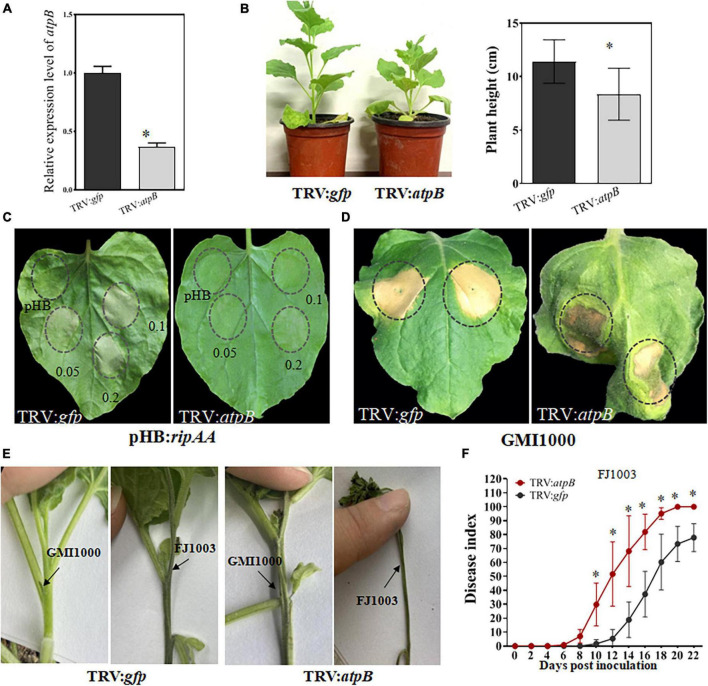
Silencing of the chloroplastic *atpB* gene in *Nicotiana benthamiana*. **(A)** Detection of the transcript level of chloroplastic *atpB* gene in gene-silenced plants. The transcript level was compared with *gfp*-silenced control plants was set to 1, and the level in *atpB*-silenced plants was calculated relative to that. Data represent the mean and standard deviation of three technical replicates (student’s *t*-test, *P < 0.05). The performance was repeated three times. **(B)** Growth phenotype of *atpB*-silenced *Nicotiana benthamiana* plants. The plant heights were calculated from 10 plants for every group of *gfp*-silencing and *atpB*-silenced (student’s *t*-test, *P < 0.05). **(C)** The loss of a macroscopic hypersensitive response phenotype in *atpB*-silenced plants induced by RipAA. The hypersensitive responses were examined by transient expression of RipAA by *Agrobacterium*-mediated infiltration. The plant leaves were infiltrated with GV3101 at three cell densities OD_600_ = 0.2, 0.1, and 0.05. The hypersensitive response phenotypes were checked 2 days post-infiltration. **(D)** Response of the *atpB*-silenced plants inoculated with wild type GMI1000. The response was scored at 5 days post-inoculation with GMI1000 (1 × 10^7^ CFU/mL). **(E)** Wilt symptoms caused by GMI1000 using the petiole inoculation method. The photos were taken 7 days post-inoculation. *Rso* strain FJ1003 that was highly virulent on tobacco was used as the control. **(F)** Disease index of *atpB*-silenced plants inoculated with the FJ1003 strain by the soil drench method. Each point represents the mean of four independent repeats with 40 plants per line. Bars indicate the standard error of the mean (student’s *t*-test, *P < 0.05).

In comparison with the TRV2:*gfp* control plants, the transcript level of chloroplastic *atpB* was reduced by 74% in *atpB*-silenced plants, indicating that the chloroplastic *atpB* gene had been successfully silenced (Figure 4A). The silencing of chloroplastic *atpB* led to a stunted phenotype with reduced plant height (Figure 4B). RipAA was transiently expressed on TRV:*atpB* plants by *Agrobacterium* infiltration with a cell density series of OD_600_ = 0.2, 0.1, and 0.05. No macroscopic HR reaction was detected from the infiltration areas in TRV:*atpB* plant leaves. In contrast, the transient expression of RipAA induced visual HR at all 3 cell density series in the TRV2:*gfp* control plants (Figure 4C). Furthermore, the infiltration of GMI1000 caused a water soaking-like phenotype in the TRV:*atpB* plant leaves, and the water soaking developed into wilt symptoms at 5 days post-inoculation (Figure 4D). To verify the wilt symptoms caused by GMI1000 on *atpB*-silenced plants, GMI1000 was inoculated on the plants using the petiole inoculation method. At 5 days post-inoculation, GMI1000 caused wilt symptoms, albeit the symptoms were weaker than those of the highly virulent *Rso* FJ1003 strain (Figure 4E). The *atpB*-silenced plants were more sensitive to *Rso*. The disease index of TRV:*atpB* plant was distinctively higher than that of the TRV2:*gfp* plant after inoculation with *Rso* FJ1003 using the soil soaking method (Figure 4F).

### 3.5. *N. benthamiana* chloroplastic AtpB shares extremely high identity with the homologs in other *Rso* hosts

**FIGURE 5:**
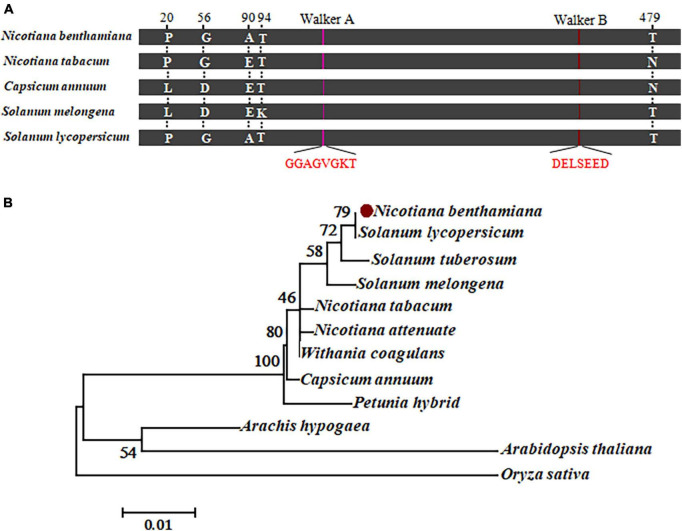
Sequence analysis of chloroplastic AtpB. **(A)** Schematic map representing the amino acid conservation in chloroplastic AtpB homologs from representative host plants. Five different residues at positions 20, 56, 90, 94, and 479 are indicated. The conserved Walker A and B motifs are highlighted in red. **(B)** Molecular phylogenetic analysis of amino acid sequences from 12 chloroplastic AtpB homologs by the maximum likelihood method. The evolutionary history is inferred based on the JTT matrix-based model. The percentage of trees in which the associated taxa clustered together is shown next to the branches. There are a total of 498 positions in the final dataset.

As a further step to understand the role of RipAA in the interaction between hosts and *Rso*, we analyzed chloroplastic AtpB homolog sequences from representative hosts of *Rso*. The homologs from *C. annuum*, *S. lycopersicum* and *S. melongena* share an extremely high identity with the chloroplastic AtpB from *N. benthamiana* and *N. tabacum*. There are a total of 5 differences in position among those homologs at 20, 56, 90, 94 and 479 across the entire 498 amino acids (Figure 5A and Supplementary Figure 2). The Walker A motif (GGAGVGKT) and a Walker B motif (DELSEED) are strictly conserved in all the chloroplastic AtpB sequences from *N. benthamiana*, *N. tabacum, S. lycopersicum* and *S. melongena* (Figure 5A). A neighbor-joining phylogenetic tree was established from the chloroplastic AtpB sequences from 12 plants that contained 10 representative hosts for *Rso*. The results showed that the chloroplastic AtpBs from nine *Rso* hosts were clustered together, including those of *N. benthamiana*, *S. lycopersicum, S. tuberosum, S. melongena, N. tabacum, N. attenuate, Withania coagulans, C. annuum*, and *Petunia hybrida*. The chloroplastic AtpBs of *Arachis hypogaea* and *A. thaliana* were grouped in another branch of trees (Figure 5B).

## 4. Discussion

The *Rso* species complex exhibits varied pathotypes on hosts (Salgon et al., 2017), which are directed by T3SS effectors (Landry et al., 2020). A number of effectors that possess putative avirulent protein domains have been identified from *Rso* based on a pan-genome analysis (Genin and Denny, 2012; Ailloud et al., 2015; Cho et al., 2019; Sabbagh et al., 2019). Among these, some are involved in the determination of host range. The RipAZ1 from strain Pe_26 and RipB from strain RS1000 are responsible for the avirulent properties on American black nightshade (*S. americanum*) and *Nicotiana* species, respectively, (Nakano and Mukaihara, 2019; Moon et al., 2021). The RipAA and RipP1 in GMI1000 were found to jointly determine an incompatible reaction on species of *Nicotiana* (Poueymiro et al., 2009). In this study, we showed that RipAA interacted with the *N. benthamiana* chloroplastic AtpB. Silencing the *atpB* gene in *N. benthamiana* led to conversion from an incompatible interaction to a compatible one with GMI1000. This suggested that the recognition between RipAA and chloroplastic AtpB is essential for the incompatible interaction with *N. benthamiana*.

The transient expression of RipAA in *N. benthamiana* induced the accumulation of H_2_O_2_ and DNA degradation, except for the visual HR. The cell death marker genes *hsr203J* and *hin1* were induced, which is consistent with these physiological changes. The enhanced expression of *PR1a* and *NPR1* suggested that RipAA-induced HR is dependent on the SA signaling pathway. In contrast, the JA signaling markers *COI1*, *MYC2*, and *PDF1.2* were significantly suppressed. The involvement of SA signaling in resistance to bacterial wilt has been clearly elucidated in several *Rso* hosts, including *S. lycopersicum*, *C. annuum*, *A. thaliana*, and *Medicago truncatula* (Chen et al., 2009; Denancé et al., 2013; Yamchi et al., 2018; Huang et al., 2020). To promote disease development, *Rso* infection induces the production of JA and simultaneously suppresses the biosynthesis of SA on susceptible *C. annuum* plants. This signaling mode is specifically regulated by the effector RipAL that targets chloroplasts (Nakano and Mukaihara, 2018). RipAA-mediated antagonistic signaling between SA and JA may promote plant resistance to *Rso* infection.

A previous study reported that RipAA does not contain large α-helices or β-sheet structures and lacks conserved motifs (Robertson et al., 2004). By using the RaptorX analysis, five α-helices and nine β-sheets were elaborately observed in RipAA. This is consistent with previous research because each of those structures did not exceed 16 amino acids. The third α-helix and ninth β-sheet contain only two amino acids. However, the predicted 3-dimensional structure showed a perfect head (mostly β-sheets), intermediate (mostly α-helices), and tail (N-and C-terminus) partitions. It appears that the tail partition composed of the N- and C-termini is absolutely required for the induction of HR in *N. benthamiana*. The 18–amino acid sequence region from positions 32 to 50 at the N-terminus is important for the HR-inducing activity (Poueymiro et al., 2009). The deletion of C-terminal 14 amino acids behind the last small β-sheet (199–200) also inactivated the ability of RipAA to induce HR. The combined evidence demonstrates the necessity of both intact N- and C-termini for HR induction.

Because RipAA induces strong cell death in *N. benthamiana*, we did not obtain the data to determine the spatial interaction between RipAA and chloroplastic AtpB *in vivo*. However, fluorescence was occasionally observed from the cell membrane, cytoplasm, and nucleus when GFP-RipAA or YFP-RipAA was transiently expressed in *N. benthamiana* at 36 h post-infiltration before macroscopic HR became apparent. The localization to different subcellular compartments after delivery into plant cells will specify the interaction with specific host targets to execute its biochemical functions (Jeon et al., 2020). For example, the transcriptional activator-like effector RipTAL localizes to the nucleus and activates the expression of shorter and more efficiently translated transcripts of arginine decarboxylase involved in the biosynthesis of polyamines (Wu et al., 2019). Thereafter, RipAA may modulate diverse targets after it has been translocated into plant cells during *Rso* infection. Even though its subcellular localization has not been elucidated, the chloroplastic AtpB may localize to diverse organelles. A study on mice indicated that mitochondrial AtpB is delivered to the cell membrane by extracellular vesicles under physiological and pathological conditions (Ha et al., 2021).

The biogenesis of chloroplastic ATPase is a complicated and highly regulated process that requires coordination between the nuclear and plastid genomes (Drapier et al., 1992). The introduction of point mutations into the translation initiation codon of the chloroplastic *atpB* gene results in decreased translation abundance in *A. thaliana*, leading to a reduction in photosynthetic electron transports, ATP synthase accumulation, and plant growth (Rott et al., 2011). In this study, the TRV-mediated VIGS of chloroplastic *atpB* in *N. benthamiana* resulted in a similar phenotype of stunted plants. Moreover, the silenced plants were more sensitive to *Rso* infection, providing direct evidence that chloroplastic AtpB correlates with plant resistance to bacterial disease.

Silencing the chloroplastic *atpB* gene in *N. benthamiana* converts an incompatible interaction to a compatible one with GMI1000. In addition to the water soaking phenotype on leaves, the wild type GMI1000 caused virtual wilt symptoms in *atpB*-silenced plants when inoculated on the petioles. This supported that the recognition between RipAA and chloroplastic AtpB results in the incompatible interaction of GMI1000 on *N. benthamiana*. However, the molecular events mediated by RipP1 merit additional study, which will facilitate global knowledge on the incompatible interaction between GMI1000 and *N. benthamiana*. The recognition between RipAA and chloroplastic AtpB may contribute to the induction of defense on a broad array of hosts because chloroplastic AtpB homologs are highly conserved in *C. annuum*, *S. lycopersicum*, and *S. melongena*, which are favorable hosts for *Rso* GMI1000. The transient expression of RipAA in *C. annuum* induced a similar hypersensitive response as in *N. benthamiana* (Supplementary Figure 3). This suggested that RipAA-induced signaling in *C. annuum* is at least partially similar to that in *N. benthamiana.*

In conclusion, our study shows that the *Rso* effector RipAA targets the chloroplastic AtpB that modulates an incompatible interaction with *N. benthamiana*. The results not only contribute to a better understanding of the molecular mechanisms by which *Rso* specifies host range but also highlight the role of chloroplastic AtpB in plant-microbe interactions.

## Data availability statement

The data presented in this study are deposited in the NBCI repository, accession number OQ786162.

## Author contributions

YM and LW performed the experiments. QX, QZ, and HZ designed and wrote the manuscript. All authors contributed to the article and approved the submitted version.

## Footnotes

1
https://solgenomics.net

2
http://raptorx.uchicago.edu

3
http://minnou.cchmc.org/

## Supplementary material

Supplementary Figure 1 Subcellular localization of YFP-RipAA in *Nicotiana benthamiana* cells. Samples were examined under a confocal microscope 36 h after agroinfiltration. All the experiments were repeated three times. The scale bar represents 50 μm.

Supplementary Figure 2 Alignment of the amino acid sequences of chloroplastic AtpB homologs. The sequences were retrieved from the Solanaceae Genomics Network (https://solgenomics.net). The positions with varied amino acids are indicated by stars. Conserved Walker A and Walker B motifs are indicated. Nb, *Nicotiana benthamiana*; Nt, *Nicotiana tabacum*; Ca, *Capsicum annuum*; Sly, *Solanum lycopersicum*; Sm, *Solanum melongena*.

Supplementary Figure 3 RipAA induced hypersensitive response on pepper plants. **(A)** Hypersensitive response induced by RipAA on pepper plants. The agrobacteria that harbored empty vector pGDGm or pGDG-RipAA were infiltrated into plants at a concentration of OD_600_ = 0.3. A hypersensitive response was recorded at 3 days post-infiltration. The gels on right showed the expression of GFP and RipAA-GFP in Western blot using α-GFP antibody. All the experiments were conducted three times. **(B)** Cell death and hydrogen peroxide accumulation were revealed by trypan blue and DAB staining. The infiltration areas are indicated by circles. GFP, green fluorescent protein; DAB, 3,3′-diaminobenzidine.

Supplementary Table 2 Primers used for molecular cloning and qRT-PCR.
