# Supplementary material for: Ralstonia solanacearum type III effector RipAA targets chloroplastic AtpB to modulate an incompatible interaction on Nicotiana benthamiana
Source: Front Microbiol. 2023 May 18;14:1179824. doi: 10.3389/fmicb.2023.1179824 (PMC10232776; doi:10.3389/fmicb.2023.1179824)
Supplement: Supplementary file 1 [file Table_1.DOCX]

Table S1. Bacterial strains and plasmids used in this study

| **Strains or plasmids** | **Relevant characteristics** | **Resources** |
| --- | --- | --- |
| **Strains** | | |
| *Ralstonia solanacearum* | | |
| GMI1000 | Wild-type, phylotype I, biovar 3, race 1 | Boucher et al., 1985 |
| FJ1003 | A wild type *Ralstonia solanacearum* strain isolated from *Nicotiana tobaccum* | Chen et al., 2022 |
| *Escherichia coli* | | |
| DH5α | *F^-^ recA hsdR17 (rk^−^, mk^+^) ϕ80lacZ∆M15* | Clontech |
| BL21 | *F^-^, ompT, hsdSB (rB^-^mB^-^), gal, dcm* | Novagen |
| *Saccharomyces cerevisiae* | | |
| AH109 | MATa, *trp1-901, leu2-3, 112, ura3-52, His3-200, gal4, gal80, LYS2::GAL1UAS-GAL1TATA-His3* | Clontech |
| *Agrobacterium tumefaciens* | | |
| GV3101 | Rif^r^, with Ti plasmid pMP90 | Koncz and Schell, 1996 |
| **Plasmids** | | |
| pHB | Km^r^, a binary vector to express gene under control of a double CaMV 35S promoter | Mao et al., 2005 |
| pHB:RipAA | Km^r^, the 693-bp full length *PopA1* gene cloned in pHB at *Hin*dIII and *Pst*I sites | This study |
| pHB:RipAA_1-216_ | Km^r^, the 648-bp fragment of *PopA1* gene cloned in pHB at *Hin*dIII and *Pst*I sites | This study |
| pHB:RipAA_1-196_ | Km^r^, the 588-bp fragment of *PopA1* gene cloned in pHB at *Hin*dIII and *Pst*I sites | This study |
| pHB:RipAA_1-166_ | Km^r^, the 498-bp fragment of *PopA1* gene cloned in pHB at *Hin*dIII and *Pst*I sites | This study |
| pHB:RipAA_1-125_ | Km^r^, the 375-bp fragment of *PopA1* gene cloned in pHB at *Hin*dIII and *Pst*I sites | This study |
| pHB:RipAA_1-89_ | Km^r^, the 267-bp fragment of *PopA1* gene cloned in pHB at *Hin*dIII and *Pst*I sites | This study |
| pET41a (+) | Km^r^, IPTG-inducible expression vector | Novagen |
| pET41:AtpB | Km^r^, full lenght *atpB* gene cloned in pET41a(+) at *Eco*RI and *Sal*I sites for expressing GST-AtpB fusion | This study |
| pMAL-4X-1 | Amp^r^, an expressing vector with maltose binding protein (MBP) tag | New England Biolabs |
| pMAL:RipAA | Amp^r^, full lenght *ripAA* gene cloned in pMAL-4X-1 at *Eco*RI and *Pst*I sites for expressing MBP-RipAA | This study |
| pGADT7 | Km^r^, SV40 NLS GAL4 AD LEU2, HAN epitope tag | Clontech |
| pGADT7:RipAA | Km^r^, 693-bp full length *ripAA* gene cloned in pGADT7 at *Nde*I and *Eco*RI sites | This study |
| pGBKT7 | Km^r^, GAL4(1–147) DNA-BD, TRP1, c-Myc epitope tag | Clontech |
| pGBKT7:RipAA | Km^r^, 693-bp full length *ripAA* gene cloned in pGBKT7 at *Nde*I and *Eco*RI sites | This study |
| pGBKT7:AtpB | Km^r^, 1497-bp full length *atpB* gene cloned in pGBKT7 at *Nde*I and *Eco*RI sites | This study |
| pGBKT7:SDE1 | Km^r^, a 465-bp coding sequence of *SDE1* cloned in pGBKT7 | Zhou et al., 2020 |
| pGADT7:NbDDX3 | Amp^r^ , the 1413-bp *NbDDX3* genes cloned in pGADT7 | Zhou et al., 2020 |
| TRV1 | Km^r^, a VIGS vector encoding the replication, movement and cysteine-rich protein protoeins of tobacco rattle virus | Liu et al., 2002 |
| TRV2 | Km^r^, a VIGS vector harboring the coat protein and two non-structural proteins of tobacco rattle virus | Liu et al., 2002 |
| TRV:*PDS* | Km^r^, a 369-bp DNA fragment of *NbPDS* cloned in TRV2 | Liu et al., 2002 |
| TRV:*gfp* | Km^r^, a 358-bp fragment of *gfp* gene cloned in TRV2 | Sun et al., 2020 |
| TRV:*atpB* | Km^r^, a 404-bp fragment of *atpB* gene cloned in TRV2 at *Xba*I and *Bam*HI sites | This study |
| pGDG | Km^r^, GFP transient expression vector | Goodin et al., 2002 |
| pGDG:RipAA | Km^r^, 693-bp coding sequence of *ripAA* cloned in pGDG at *Bgl* Ⅱ and *Sal* I sites | This study |
| pGD-3G-mCherry | Km^r^, mCherry transient expression vector | Sun et al., 2018 |
| pGD3G:RipAA | Km^r^, 693-bp coding sequence of *ripAA* cloned int pGD3G-mCherry at *Xho*I and *Sal* I sites | This study |
| pICH47811 | Km^r^, YFP transient expression vector | Lab collection |
| pICH47811:RipAA | Km^r^, 689-bp coding sequence of *ripAA* cloned in pICH47811 fused with YFP | This study |

**References**

Boucher, C.A., Barberis, P.A., Trigalet, A.P., Demery, D.A. (1985). Transposon mutagenesis of *Pseudomonas solanacearum*: isolation of Tn5-induced avirulent mutants. *J. Gen.Microbiol.* 131: 2449–2457.

Goodin, M.M., Dietzgen, R.G., Schichnes, D., Ruzin, S., Jackson, A.O. (2002). pGD vectors: versatile tools for the expression of green and red fluorescent protein fusions in agroinfiltrated plant leaves. *Plant J.* 31: 375–383.

Koncz, C., Schell, J. (1986). The promoter of TL-DNA gene 5 controls the tissuespecific expression of chimeric genes carried by a novel type of *Agrobacterium* binary vector. *Mol. Gen. Genet.* 204: 383–396.

Liu, Y., Schiff, M., Dinesh-Kumar, S.P. (2002). Tobacco *Rar1*, *EDS1* and *NPR1/NIM1* like genes are required for N-mediated resistance to tobacco mosaic virus. *Plant J.* 30: 415-429.

Mao, J., Zhang, Y.C., Sang, Y., Li, Q.H., Yang, H.Q. (2005). A role for *Arabidopsis* cryptochromes and COP1 in the regulation of stomatal opening. *Proc. Natl. Acad. Sci. USA* 102: 12270–12275.

Sun, Q., Li, Y.Y., Wang, Y., Zhao, H.H., Zhao, T.Y., Zhang, Z.Y., Li, D.W. et al. (2018). Brassica yellows virus P0 protein impairs the antiviral activity of NbRAF2 in *Nicotiana benthamiana*. *J. Exp. Bot.* 69: 3127-3139.

Sun, T., Wu, W., Wu, H., Rou, W., Zhou, Y., Zhuo, T., et al. (2020) *Ralstonia solanacearum* elicitor RipX induces defense reaction by suppressing the mitochondrial *atpA* Gene in host plant. *Int. J. Mol. Sci.* 21: 2000.

Chen K, Zhuang Y, Wang L, Li H, Lei T, Li, M Gao, M, Wei J, Dang H, Raza A, et al. (2022). Comprehensive genome sequence analysis of the devastating tobacco bacterial phytopathogen *Ralstonia solanacearum* strain FJ1003. *Front Genet.* 13: 966092.

Zhou, Y., Wei, X., Li, Y., Liu, Z., Duan, Y., Zou, H. (2020). '*Candidatus* liberibacter asiaticus' SDE1 effector induces Huanglongbing chlorosis by downregulating host *DDX3* Gene. *Int. J. Mol. Sci.* 21: 7996.
