# Supplementary material for: Ralstonia solanacearum type III effector RipAA targets chloroplastic AtpB to modulate an incompatible interaction on Nicotiana benthamiana
Source: Front Microbiol. 2023 May 18;14:1179824. doi: 10.3389/fmicb.2023.1179824 (PMC10232776; doi:10.3389/fmicb.2023.1179824)
Supplement: Supplementary file 2 [file Table_3.docx]

Table S3 Protein candidates identified from *N. benthamiana* through co-immunoprecipitation

| **Accession** | **Description** | **Mass** | **Score** | **Coverage** | **Localization** |
| --- | --- | --- | --- | --- | --- |
| P69369 | ATP synthase β subunit | 53511 | 3610 | 60% | chloroplastic |
| P48709 | Ribulose bisphosphate carboxylase large chain | 54872 | 4573 | 47% | chloroplastic |
| P00823 | ATP synthase subunit α | 55410 | 2386 | 30% | chloroplastic |
| Q42961 | Phosphoglycerate kinase | 51037 | 2330 | 49% | chloroplastic |
| P27141 | Carbonic anhydrase | 30610 | 1082 | 45% | chloroplastic |
| P09043 | Glyceraldehyde-3-phosphate dehydrogenase A | 42122 | 1284 | 35% | chloroplastic |
| Q40460 | Ribulose bisphosphate carboxylase/oxygenase activase 1 | 48951 | 2011 | 46% | chloroplastic |
| Q40565 | Ribulose bisphosphate carboxylase/oxygenase activase 2 | 48541 | 1878 | 45% | chloroplastic |
