# Supplementary figures and images for: Ralstonia solanacearum type III effector RipAA targets chloroplastic AtpB to modulate an incompatible interaction on Nicotiana benthamiana
Source: Front Microbiol. 2023 May 18;14:1179824. doi: 10.3389/fmicb.2023.1179824 (PMC10232776; doi:10.3389/fmicb.2023.1179824)

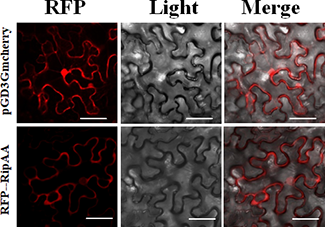

Supplement: Supplementary Figure 1 — Subcellular localization of YFP-RipAA in Nicotiana benthamiana cells. Samples were examined under a confocal microscope 36 h after agroinfiltration. All the experiments were repeated three times. The scale bar represents 50 μm. [file Image_1.TIF]

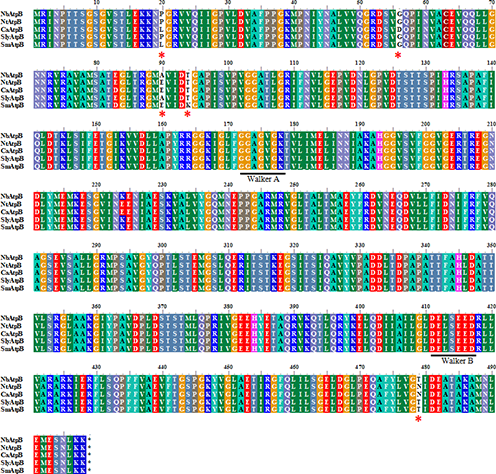

Supplement: Supplementary Figure 2 — Alignment of the amino acid sequences of chloroplastic AtpB homologs. The sequences were retrieved from the Solanaceae Genomics Network (https://solgenomics.net). The positions with varied amino acids are indicated by stars. Conserved Walker A and Walker B motifs are indicated. Nb, Nicotiana benthamiana; Nt, Nicotiana tabacum; Ca, Capsicum annuum; Sly, Solanum lycopersicum; Sm, Solanum melongena. [file Image_2.TIF]

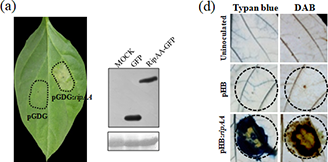

Supplement: Supplementary Figure 3 — RipAA induced hypersensitive response on pepper plants. (A) Hypersensitive response induced by RipAA on pepper plants. The agrobacteria that harbored empty vector pGDGm or pGDG-RipAA were infiltrated into plants at a concentration of OD600 = 0.3. A hypersensitive response was recorded at 3 days post-infiltration. The gels on right showed the expression of GFP and RipAA-GFP in Western blot using α-GFP antibody. All the experiments were conducted three times. (B) Cell death and hydrogen peroxide accumulation were revealed by trypan blue and DAB staining. The infiltration areas are indicated by circles. GFP, green fluorescent protein; DAB, 3,3′-diaminobenzidine. [file Image_3.TIF]
