# Supplementary material for: Ralstonia solanacearum type III effector RipAA targets chloroplastic AtpB to modulate an incompatible interaction on Nicotiana benthamiana
Source: Front Microbiol. 2023 May 18;14:1179824. doi: 10.3389/fmicb.2023.1179824 (PMC10232776; doi:10.3389/fmicb.2023.1179824)
Supplement: Supplementary Table 2 — Primers used for molecular cloning and qRT-PCR. [file Table_2.DOCX]

| **Primer name** | **Sequence(5'to3')** | | **Products size (bp)** | **Enzyme site** | **Description or purpose** | |
| --- | --- | --- | --- | --- | --- | --- |
| **Molecular cloning** | | | | | | |
| pHBRipAA.F | TCAAGCTTATGAGAAGAATCGGCAAC | | 693 | *Hind* Ⅲ | Coding sequence of *ripAA* cloned into pHB for transient expression in *N. benthamiana* | |
| pHBRipAA.R | TACTGCAGCTAGCCGTCGCTATCGCT | |  | *Pst*Ⅰ |  |  |
| pHBRipAA1.R | TACTGCAGCTAGTTCCCCATCTGATCGG | | 648 | *PstⅠ* |  |  |
| pHBRipAA2.R | TACTGCAGCTACCCGAAAACACTCCGGC | | 588 | *PstⅠ* |  |  |
| pHBRipAA3.R | TACTGCAGCTAGTTCTGCCAATCCATGAAC | | 498 | *PstⅠ* |  |  |
| pHBRipAA4.R | TACTGCAGCTAGCCTGTCACGCTGAAGTACTG | | 375 | *PstⅠ* |  |  |
| pHBRipAA5.R | TACTGCAGCTAGAGGTCGGGGTAGCGCT | | 267 | *PstⅠ* |  |  |
| pGDGRipAA.F | GAAGATCTATGAGAAGAATCGGCAAC | | 690 | *Bgl* Ⅱ | Cloning *ripAA* in pGDG vector to express GFP-RipAA in *N. benthamiana* | |
| pGDGRipAA.R | GCGTCGACGCCGTCGCTATCGCTATC | |  | *Sal* Ⅰ |  |  |
| pmCRipAA.F | CCGCTCGAGATGAGAAGAATCGGCAAC | | 690 | *Xho*Ⅰ | Cloning *ripAA* in pGD-3G-mCherry to express GFP-RipAA in *N. benthamiana* | |
| pmCRipAA.R | GCGTCGACGCCGTCGCTATCGCTATC | |  | *Sal* Ⅰ |  |  |
| RipAA-Bsa1-F | atggtctcaaATGAGAAGAATCGGCAACT | | 689 | *-* | Cloning *ripAA* in pICH47811 to express YFP-RipAA in *Arabidopsis* protoplast | |
| RipAA-Bsa1-R | atggtctcacgaaCCGTCGCTATCGCTATCG | |  | *-* |  |  |
| ADRipAA.F | CCCATATGAGAAGAATCGGCAACT | | 693 | *Nde*Ⅰ | Cloning *ripAA* in pGADT7 for Y2H analysis | |
| ADRipAA.R | GGAATTCCTAGCCGTCGCTATCGCT | |  | *Eco*RⅠ |  |  |
| BDatpB.F | CCCATATGATGCGAATCAATCCTACTACT | | 1497 | *Nde*I | Cloning *atpB* in pGBKT7 for Y2H analysis | |
| BDatpB.R | CGGAATTCTCATTTCTTCAAATTGCTCTC | |  | *Eco*RⅠ |  |  |
| MBP-RipAA.F | gagggaaggatttcagaattcATGAGAAGAATCGGCAACTTTTTC | | 693 | *Eco*RⅠ | Cloning *ripAA* in pMAL-4X-1 for expression of MBP-RipAA | |
| MBP-RipAA.R | cagtgccaagcttgcctgcagCTAGCCGTCGCTATCGCTATCG | |  | *Pst*Ⅰ |  |  |
| PET41a-atpB.F | GGAATTCATGCGAATCAATCCTACTACT | | 1497 | *Eco*RⅠ | Cloning *atpB* in pET41a for expression of GST-AtpB | |
| PET41a-atpB.R | GCGTCGACTTTCTTCAAATTGCTCTCCAT | |  | *Sal* Ⅰ |  |  |
| TRVatpB.F | GCTCTAGAGACTCTGGGACGAATT | | 404 | *Xba*Ⅰ | Cloning a 404-bp fragment of *atpB* gene cloned in TRV2 vector for gene silencing | |
| TRVatpB.R | CGGGATCCTCATCTGACCGTAAACT | |  | *Bam*HⅠ |  |  |
| **For qRT-PCR** | | | | | | |
| RipAA.F/RipAA.R | | GACAAGCGGCTGGGAATACA/GTGCTGGTCGGGATAAACAT | | | | *ripAA* expression level |
| atpB.F/atpB.R | | AATGCCTTCCGCTGTGGGTTA/AGGGTCGGTCAAATCGTCTGC | | | | *atpB* expression level |
| NbCOI1.F/NbCOI1.R | | GAGAGGAGAGAATAACAGATC/AGCAGCCTCTCACTTCTAGC | | | | JA signaling response |
| NbMYC2.F/ NbMYC2.R | | GAAAAGAGGCCAAAGAAGCGAGGAA/CTTCAGCTCATTAATATATGAAATT | | | | JA signaling response |
| NbPDF1.2.F/ NbPDF1.2.R | | CTATGCACTAAGCCATGTGTGTTTG/CTTCAAGCAAAGCTGCAGCCAAAG | | | | JA signaling response |
| NbHIN1.F/ NbHIN1.R | | CTGCAACCCATGTAGCTGTCTC/TTTGTTAGGACGAAGAACGAGCC | | | | HR response |
| Nbhsr203J.F/ Nbhsr203J.R | | TGGCTCAACGATTACGCAGAT/GACGGCAACTTGGTGGACTA | | | | HR response |
| NbPR1a.F/ NbPR1a.R | | ATGGTCAATACGGCGAAAAC/CCTAGCACATCCAACACGAA | | | | SA signaling response |
| NbNPR1.F/ NbNPR1.R | | TAGCGTATTGCGATGCAAAG/ TAGTGAGCCTCTTGGCGATT | | | | SA signaling response |
| NbEF1α.F/NbEF1α.R | | TGGTGTCCTCAAGCCTGGTATGGTTG/ACGCTTGAGATCCTTAACCGCAACATTCTT | | | | Internal control |
